# The behaviour of stacking fault energy upon interstitial alloying

**DOI:** 10.1038/s41598-017-11328-4

**Published:** 2017-09-11

**Authors:** Jee-Yong Lee, Yang Mo Koo, Song Lu, Levente Vitos, Se Kyun Kwon

**Affiliations:** 1 Graduate Institute of Ferrous Technology, Pohang University of Science and Technology, Pohang, 37673 Korea; 2 Department of Materials Science and Engineering, Pohang University of Science and Technology, Pohang, 37673 Korea; 3 Applied Materials Physics, Department of Materials Science and Engineering, Royal Institute of Technology, Stockholm, SE-100 44 Sweden; 4 Department of Physics and Astronomy, Division of Materials Theory, Uppsala University, Box 516, SE-75120 Uppsala, Sweden; 5 Institute for Solid State Physics and Optics, Wigner Research Centre for Physics, H-1525 Budapest, P.O. Box 49, Hungary

## Abstract

Stacking fault energy is one of key parameters for understanding the mechanical properties of face-centered cubic materials. It is well known that the plastic deformation mechanism is closely related to the size of stacking fault energy. Although alloying is a conventional method to modify the physical parameter, the underlying microscopic mechanisms are not yet clearly established. Here, we propose a simple model for determining the effect of interstitial alloying on the stacking fault energy. We derive a volumetric behaviour of stacking fault energy from the harmonic approximation to the energy-lattice curve and relate it to the contents of interstitials. The stacking fault energy is found to change linearly with the interstitial content in the usual low concentration domain. This is in good agreement with previously reported experimental and theoretical data.

## Introduction

Face-centered cubic (fcc) and hexagonal close-packed (hcp) structures can be characterized by an ordered stacking of 2D honeycomb-type layers. As a consequence, the stacking fault in an fcc structure refers to the locally irregular stacking sequence along the [111] direction, which is generated by the splitting of a perfect dislocation into two Shockley partial dislocations during plastic deformation. The excess stuctural energy induced in a material by the process defines the so-called stacking fault energy (SFE, ${\gamma }_{{\rm{sf}}}$). Because a stacking fault locally resembles an hcp structure embedded in the fcc structure, the SFE can be found in relation to the energy difference between the two structures. The SFE is regarded as one of the most important parameters for understanding the mechanical properties of fcc materials.

It is an empirical rule that as the SFE value increases, the deformation mechanism of the fcc materials varies from *γ*/*ε* phase transformation to twin formation, and slip. For example, the plastic deformation behaviour of Co-Ni-Cr-Mo alloys changes from phase transformation to twinning for ${\gamma }_{{\rm{sf}}} \sim 18\,m{\rm{J}}/{{\rm{m}}}^{2}$ and to slip for $\sim 30\mbox{--}47\,m{\rm{J}}/{{\rm{m}}}^{2}$. These boundaries become ${\gamma }_{{\rm{sf}}} \sim 12\,m{\rm{J}}/{{\rm{m}}}^{2}$ and $\sim 18\mbox{--}42\,m{\rm{J}}/{{\rm{m}}}^{2}$, respectively, for Fe-Mn-Cr-C alloys^1, 2^. Similarly, transformation-induced plasticity and twinning-induced plasticity steels are classified^3^ by the critical value of ${\gamma }_{{\rm{sf}}} \sim 16\,m{\rm{J}}/{{\rm{m}}}^{2}$.

Although a considerable number of works have measured and predicted SFEs for fcc materials, the effect of alloying on the behaviour of SFE is not yet clearly understood from basic physical parameters. Specifically, the influence of interstitial elements on the SFE of fcc materials such as austenitic steels is usually quite significant, but it remains vague for some cases. From the Hume-Rothery rules^4^, a solid solution will not be substitutional when the difference in the atomic radii of the solute and solvent atoms is more than 15%. Therefore, we can generally regard carbon and nitrogen as the interstitial elements because their atomic sizes are significantly smaller than those of the metallic elements which form the crystalline lattice, and are comparable to the size of interstices.

The stacking fault energy values measured for Fe-18Cr-10Mn-0.2Si-0.06C-N^5^ and Fe-15Mn-2Cr-0.6C-N^6^ alloys using neutron diffraction (ND) and X-ray diffraction (XRD) methods, respectively, increased linearly with interstitial content. These overall increasing trends were also demonstrated with transmission-electron microscopy (TEM) measurements for Fe-12Mn-C, Fe-18Mn-10Ni-C and Fe-18Cr-16Ni-10Mn-N alloys, while a decreasing SFE behaviour was locally observed for Fe-22Mn-C and Fe-15Cr-17Mn-N in the low carbon and nitrogen regions^7, 8^.

In addition, recent *ab-initio* calculations also exhibit the linear tendency of SFE with interstitials. Non-magnetic *γ*-Fe shows enhanced SFE values with the homogeneous alloying of carbon and nitrogen, and the effect of nitrogen is also similar in the Fe-12Mn-N system^9, 10^. Moreover, magnetic and chemical disorders in austenitic Fe-Cr-Ni stainless steels retain linear SFE behaviour with the addition of interstitial carbon^11^. Hence, interstitial elements might be regarded as inducing the linear trend in SFE. In this work, we provide the origin of the physical properties and its implications.

## Results and Discussion

Using the structural characteristic of a stacking fault, Olson and Cohen^12^ suggested a formulation for SFE, as

1 $${\gamma }_{{\rm{sf}}}=2\rho {\rm{\Delta }}{G}^{\mathrm{hcp}-\mathrm{fcc}}+2\sigma ,$$

where ${\rm{\Delta }}{G}^{\mathrm{hcp}-\mathrm{fcc}}$ is the free energy difference between the hcp and fcc structures, $\rho$ is the surface atomic number density of the (111) layer, and $\sigma$ is the fcc/hcp interfacial free energy. The factor of 2 reflects the two interfaces between the fcc structure and stacking fault. The variation in $\sigma$ with composition, temperature, and volume is often neglected for the same kind of materials^13^.

It is a first principle in physics that the response of a material in equilibrium to external perturbation appears to be the second order of the variation of the physical parameter. By employing this fact, we expand the total energy of a given material near equilibrium as a function of volume $V$. It is straightforward to obtain the energy difference of the hcp and fcc structures

2 $${E}_{h}(V)-{E}_{f}(V)\approx \{{E}_{h}({V}_{0})-{E}_{f}({V}_{0})\}+{B}_{h}(\frac{{V}_{0}-{V}_{1}}{{V}_{1}})(V-{V}_{0}),$$

where ${V}_{0}$ and ${V}_{1}$ are the fcc and hcp volumes in each equilibrium, ${E}_{f}$ and ${E}_{h}$ are the fcc and hcp total energy, respectively. The hcp bulk modulus is given by ${B}_{h}={{V}_{1}{\partial }^{2}{E}_{h}/\partial {V}^{2}|}_{{V}_{1}}$. We divide the left and right sides of equation (2) by the surface area in order to find the stacking fault energy as a function of the lattice parameter

3 $${\gamma }_{{\rm{sf}}}\,(a)={\gamma }_{{\rm{sf}}}\,({a}_{0})+\alpha \times (a-{a}_{0}),$$

where ${\gamma }_{{\rm{sf}}}\,(a)$ and ${\gamma }_{{\rm{sf}}}\,({a}_{0})$ are the SFEs of a material at the fcc volumes of $a=\sqrt[3]{V}$ and ${a}_{0}=\sqrt[3]{{V}_{0}}$, respectively, and the linear coefficient is obtained to be $\alpha =2\sqrt{3}{B}_{h}\{({V}_{0}-{V}_{1})/{V}_{1}\}$. It is clear that the SFE varies linearly in proportion to the lattice deviation from that of equilibrium ${\rm{\Delta }}a=a-{a}_{0}$. Note that the SFE and lattice parameter relationship proposed by equation (3) is consistent with equation (1) because the interfacial energy term is nearly constant and implicitly included in both sides of equation (3).

Next, we consider the effect of interstitial alloying on material volume. As far as the interstitial distribution is homogeneous and its content is low, it is sound to assume that each interstitial atom contributes an equal amount of additional volume to the host material. Thus, we write the volume change induced by a single interstitial atom as ${v}_{i}=V(N,1)-V(N,0)$, where *V*(*N*, *n*) is the volume of the material, consisting of *N* host atoms and *n* interstitial atoms. Because the number of interstitial atoms is usually small compared with that of host atoms, *n* ≪ *N*, the lattice parameter *a*(*n*) can be written as

4 $$a(n)=a(0)\{1+\frac{1}{3}(\frac{n}{N})(\frac{{v}_{i}}{{v}_{0}})\},$$

where is defined as the initial material volume per atom. After noticing that *n*/*N* is related to the interstitial concentration $c=n/(N+n)$, the above equation can also be expressed as

5 $$a(c)=a(0)+\beta \times \frac{c}{1-c},$$

with the constant $\beta =({a}_{0}/3)({v}_{i}/{v}_{0})$

Hence, the internal pressure effect of interstitials on SFE can be obtained by combining equations (3) and (5),

6 $${\gamma }_{{\rm{sf}}}\,(c)={\gamma }_{{\rm{sf}}}\,(0)+k\times \frac{c}{1-c},$$

where $k=\alpha \beta$ is understood to be the characteristic constant of a given material, to represent the change in SFE produced by interstitial alloying. Interstitial content in a material usually does not exceed a few percent. Thus we may set $c \sim {\mathscr{O}}\,({10}^{-2})$ and derive ${\gamma }_{{\rm{sf}}}\,(c)={\gamma }_{{\rm{sf}}}\,(0)+kc$. It is remarkable that the stacking fault energy linearly depends upon the interstitial concentration in the low *c* regime.

**Figure 1:**
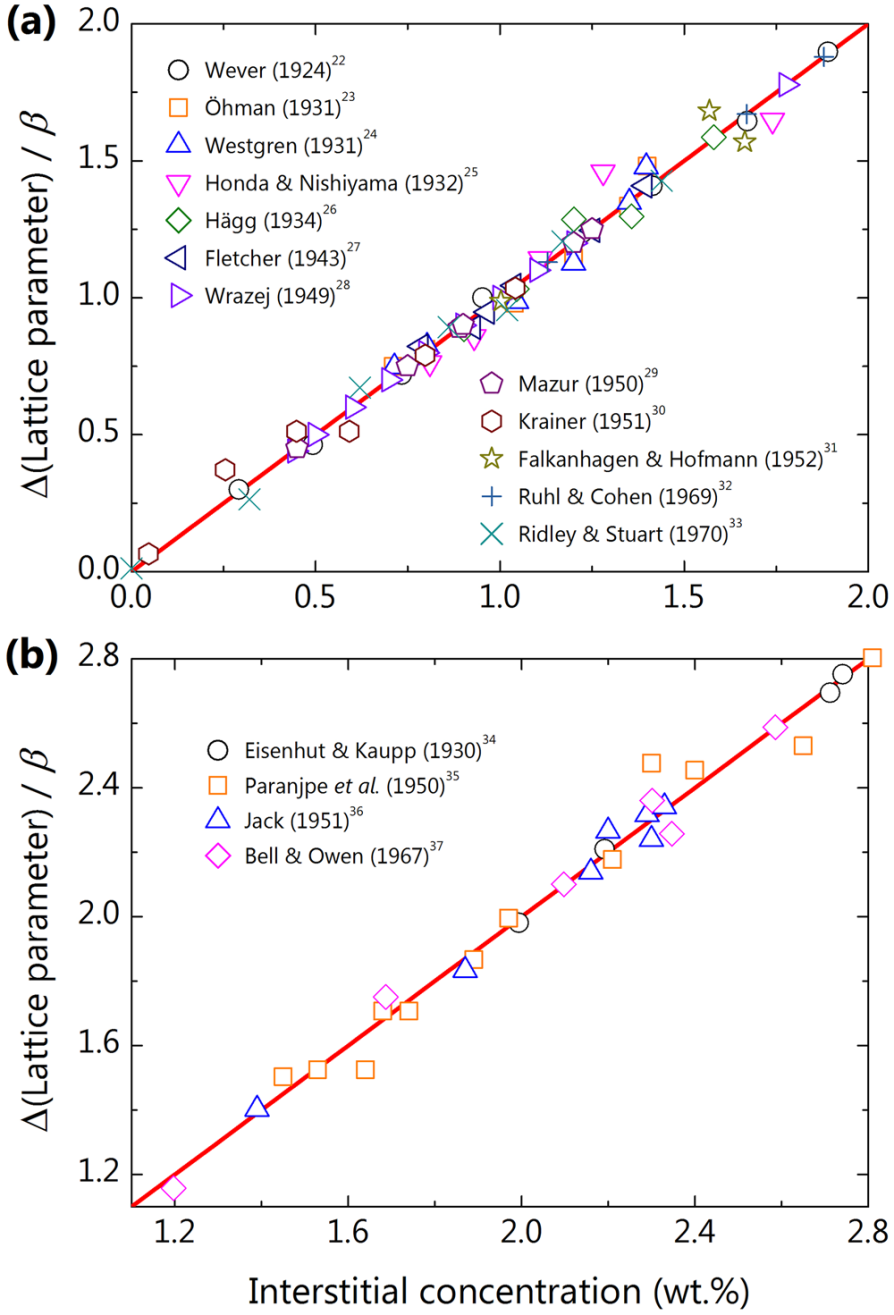
The behaviour of lattice parameter with interstitial concentration in the Fe-C and Fe-N binary alloys. The experimental data were compiled from the literature. Both the Fe-C (**a**) and Fe-N (**b**) alloys clearly show a linear variation in lattice with increasing interstitial alloying.

To examine the reliability of the proposed relationships, in Fig. 1 we plot the lattice parameter versus interstitial concentration with the known data for the Fe-C and Fe-N binary alloys. The lattice parameters were adjusted to zero for $a(0)$ and scaled by the proportional coefficient $\beta$ in equation (5). Clearly, both the carbon (Fig. 1(a)) and nitrogen (Fig. 1(b)) linearly increase the lattice constant as their contents increase.

**Figure 2:**
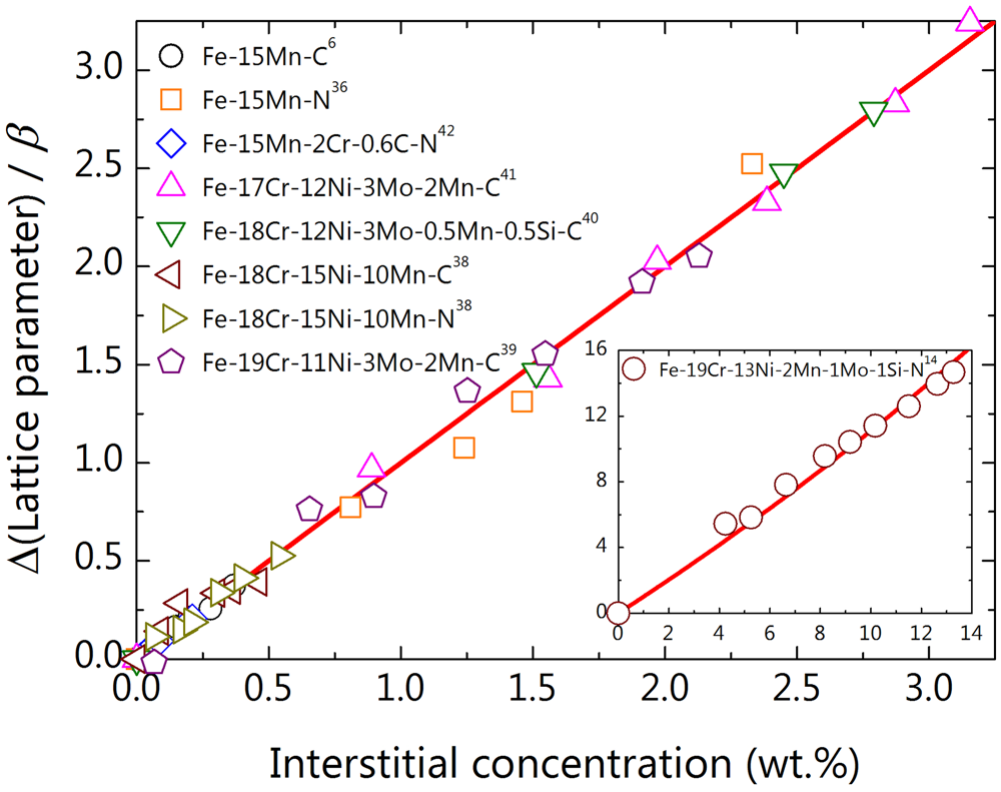
The behaviour of lattice parameter with interstitial concentration in multi-component alloys. The experimental data are for austenitic steels from the literature. All data consitently fit to the theoretical linear line at low interstitial concentrations. As shown in the inset with the Fe-19Cr-13Ni-2Mn-1Mo-1Si-N alloy, the non-linear behaviour of lattice parameter at high interstitial concentration is also well-captured by the present results.

Like the cases of the Fe-C and Fe-N alloys, in Fig. 2 we compare the lattice parameter versus interstitial concentration relationship with previously reported experimental data for austenitic steels. It is evident that all the experimental data in the considered multi-component alloys follow the current theoretical linear line well.

The present result demonstrated in Figs 1 and 2 can be regarded in a mathematical sense as a simple linear expansion of the lattice parameter near a small alloy concentration. However, we provided a physical meaning to the expansion coefficient $\beta =({a}_{0}/3)({v}_{i}/{v}_{0})$ in equation (5) behind the abstract mathematical treatment. We can estimate ${v}_{i}/{v}_{0} \sim 1/2$ for carbon in austenitic steels, which is large and not at all obvious by intuition. In addition, the lattice-interstitial concentration relationship of equation (5) can be applied to the extended range of interstitial concentration. For example, the inset in Fig. 2 shows the lattice parameter behaviour for high interstitial concentration with the experimental data of Fe-19Cr-13Ni-2Mn-1Mo-1Si-N^14^. When one tries a linear fit to the displayed data, it suffers from substantial deviation when extrapolated to the low concentration region. In contrast, the curve from equation (5) shown in the inset behaves properly through the whole considering range of interstitial concentration. Therefore, the empirical assumption of a linear relationship between the lattice parameter and interstitial alloying at low concentration^15–17^ is clearly resolved, and can be extended to the higher concentration region.

**Figure 3:**
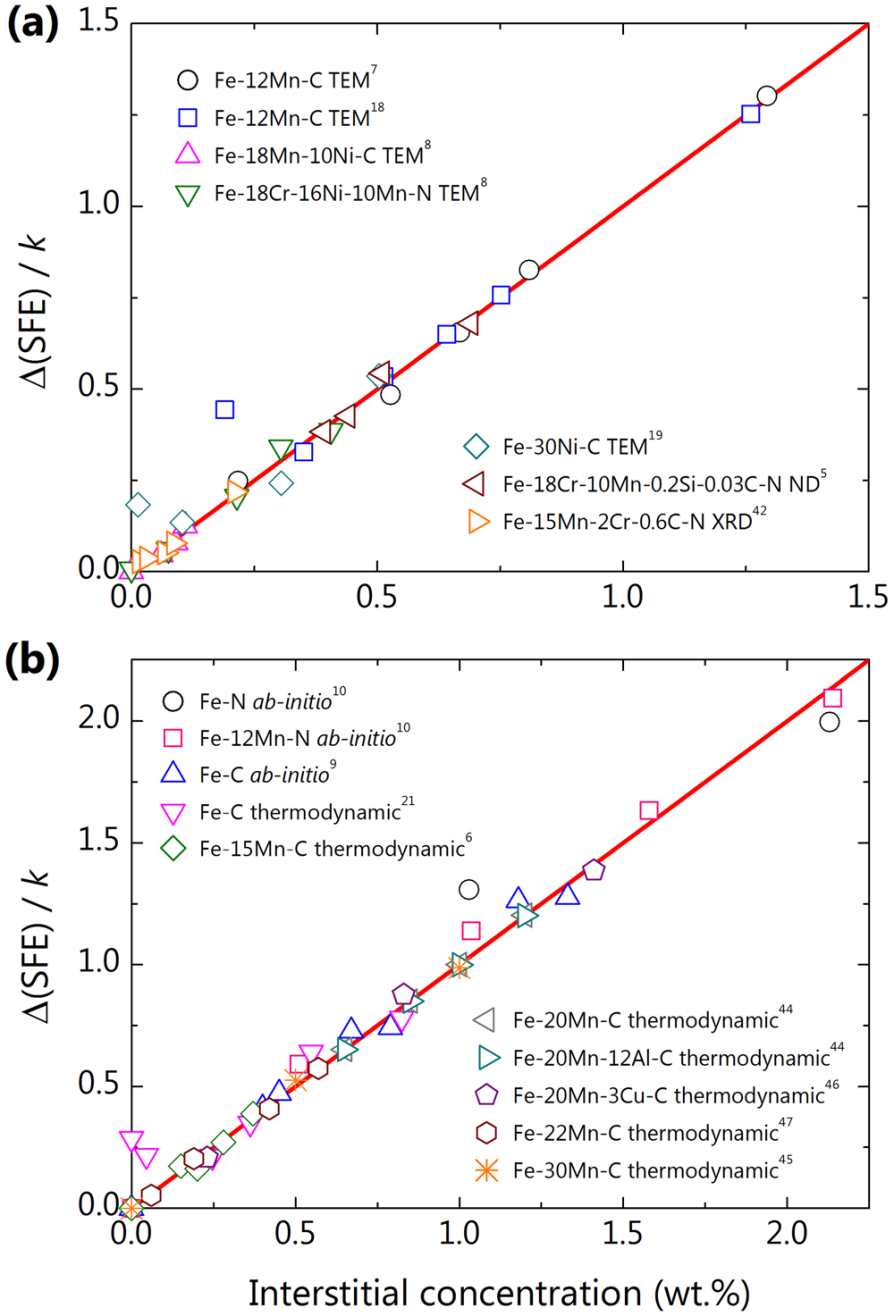
The behaviour of stacking fault energy with interstitial concentration. Data shown are for some austenitic steels from experiments (**a**) and theoretical calculations (**b**), respectively, in the literature. Clearly, all data well follow a linear line within the range of conventional interstitial concentrations. The weak deviations from the linear line observed in some experiments come from extrinsic factors, as discussed in the main text.

Figure 3 shows the change in SFE as a function of interstitial concentration with experimental and theoretical data for austenitic steels found in the literature. The SFEs are scaled using equation (6) in a process similar to the lattice parameter in Figs 1 and 2. It is very interesting that the SFE increases as the interstitial concentration increases. Both the experimental (Fig. 3(a)) and theoretical (Fig. 3(b)) data consistently support the linear behaviour of SFE variation in relation to interstitial content at low concentration.

Although a weak anomaly is locally observed in the TEM-measured SFE of Fe-12Mn-C^18^ and Fe-30Ni-C^19^ alloys in very low concentrations, this does not appear in another TEM measurement with one of the same Fe-12Mn-C alloys^7^. As suggested by Hickel *et al*., these phenomena might arise as an effect due to local heating of the specimen during TEM measurement^20^. Their *ab-initio* calculations indicated that interstitial carbons in the central layer of a stacking fault sharply increase SFE, and thus enhanced carbon diffusivity near the heated zone may alter the SFE of specimen. Therefore, it is likely that the local deviations from the linearity in some of the TEM-measured SFE data are extrinsic effects that depend specifically on the specimen temperature during measurement. The SFE trends in the TEM measurements of the Fe-22Mn-C and Fe-15Cr-17Mn-N alloys^8^ can also be understood in a similar way. Note that the SFE values obtained by other diffraction methods are highly consistent with our observation of linearity.

Theoretical calculations in Fig. 3(b) provide additional support to the current model of the effect of interstitial alloying on SFE. All data sets in Fig. 3(b) are closely bound to the ideal curve from equation (6). The small deviation in the Fe-C binary alloy^21–47^ is attributed to errors in the experimental data that was used to build the thermodynamic model. It is an important fact that the trend in SFE with respect to interstitials does not rely on the details of the substitutional alloy composition. This observation might imply weak chemical interactions between the interstitial and constituent metallic atoms inside the materials. It was previously reported that the formation of a stacking fault tends to deplete interstitial atoms at the structure, which reduces the SFE by about one-third^20^. Another *ab-initio* study showed that for the paramagnetic *γ*-Fe and the Fe-20Cr-8.4Ni alloy, at least 70% of the change in SFE produced by interstitial carbons relies on the volumetric effect^11^. Therefore, the leading term in the variation of SFE with interstitial alloying can be considered to have a mechanical origin rather than reflecting a chemical effect.

Once again, we would like to emphasize that equation (6) is an accurate description of the SFE response to interstitial contents. It implicitly incorporates the interfacial energy term, which is higher order and less variant. When a chemical effect needs to be considered, within the weak interaction regime, one can formally adopt the same form of the relationship with a slightly modified coefficient constant $k$ in equation (6). It is also expected that a similar idea can be applied to the cases of substitutional alloys within the low concentration domain, where the concentration dependency will be given by $c/(1-c)\to c$. The non-linear form of the SFE with respect to interstitials in equation (6) naturally guarantees extended validity to the higher concentration regime. Furthermore, the newly introduced physical parameters $\alpha$ and $\beta$ are expressed in terms of well-defined material properties like bulk modulus and lattice constant/volume and no other arbitrary parameters are used. Thus, the present discussions are also proper from the viewpoint of theoretical development.

In conclusion, we established a physical model of the SFE change induced by interstitial alloying and showed that the volumetric effect gives rise to the dominant behaviour of the SFE. The derived relationship is mostly suitable for a low concentration of interstitials, but encompasses the usual range of alloy concentrations in materials. We are convinced that the present model will find an application in alloy deign for engineering materials.
